# Mixed methods realist evaluation of a co-designed intervention to promote shared decision-making with frail older adults, planning discharge from hospital: a protocol

**DOI:** 10.1186/s12877-025-06581-6

**Published:** 2025-11-20

**Authors:** Kerrie McLarnon, Peter O’Halloran, Deirdre McGrath, Christine Brown Wilson

**Affiliations:** School of Nursing and Midwifery, Queen’s University Belfast, Medical Biology Centre, 97 Lisburn Road, Belfast, BT9 7BL Northern Ireland

**Keywords:** Hospital discharge, Shared decision-making, Older people, Frailty, Realist review

## Abstract

**Background:**

Effective planning for hospital discharge can best be achieved through shared decision-making (SDM): providing tailored, person-centred, strengths-based choice to allow for individuals to make fully informed decisions, with input from their relatives if they wish. Decisions such as care management, emergency planning and caregiver involvement. However, SDM is challenging for healthcare professionals and patients, particularly older people living with frailty. There is currently no consensus on the optimum way to achieve SDM, as the quality of evidence for interventions is minimal.

**Aim:**

This protocol outlines a project that aims to co-design a multi-component intervention to prepare health professionals, patients, and patients’ carers to engage in SDM and to evaluate the feasibility of implementing the intervention using realist methods.

**Methods:**

The research will be conducted in a health and social care trust in Northern Ireland with frail, older adults who are planning their discharge from hospital. The project will proceed in three stages. (1) Realist review of the literature will enable formulation of initial programme theory, including the impact of context and the identification of uncertainties. (2) Co-design of the intervention will ensure the inclusion of diverse stakeholders in the development of the intervention, with further development of the programme theory. (3) Implementation and feasibility testing of implementation processes, measurement of intervention costs, testing of outcome measures, and refinement of the programme theory.

**Outcomes:**

In line with MRC guidance for complex interventions we will seek insights to help refine the SDM intervention. The overall outcome evaluation will be guided by the ‘Quintuple Aim’ framework, which has five aims of seeking to advance health equity, enhance patient experience, improve health, reduce costs, and improve the work life of healthcare professionals.

**PROSPERO:**

(International Prospective Register of Systematic Reviews) registration number CRD42024541455.

**Clinical trial number:**

Not applicable.

## What is the problem being addressed?

Shared decision-making (SDM) is in use across multiple healthcare settings [1] and is embedded in health policy at international level. However, at an operational level, patients are not always recognised as active participants [1] despite the traction SDM is gaining globally. SDM is a cornerstone of person-centred care thus it is imperative that we seek to understand why it is so difficult to implement in practice.

Shared decision-making (SDM) has been defined as ‘a collaborative process that involves a person and their healthcare professional working together to reach a joint decision about care, now or in the future’ [2]. It involves healthcare professionals working together with people who use services and their families and carers to choose tests, treatments, management or support packages, based on evidence and informed personal preferences, health beliefs, and values’ [2]. However, as Naik et al. [3] have highlighted, older persons bring complex and highly individualised medical, functional, and social problems to their clinical encounters, and often have perceptions and preferences for care that diverge or conflict with their clinicians’ preferences [4–7].

The National Institute for Health and Care Excellence (NICE) recommends that SDM be made part of everyday care in all healthcare settings [8]. While no universal definition exists, models of SDM interventions typically seek to create choice awareness, describe treatment options, tailor information, elicit patient preferences, and enable decision making [1]. Various modalities are used, including educational meetings and written materials, audit and feedback, audio-visual materials, video modelling, role playing, group and individual approaches, and decision-support tools [9]. The evidence suggests treating SDM as an ongoing process and using multicomponent rather than individual interventions is likely to be most effective; [8] and that patients’ preparedness for SDM increases their confidence to take responsibility for their treatment decisions and their involvement in SDM [10]. Interventions to promote SDM can be divided into three categories: those that train healthcare professionals; those for patients only; and those that are aimed at both professionals and patients [11]. Some are focused on specific healthcare settings and patient groups, whilst others are more generic [1].

Hospital discharge of older patients is a high-risk situation in terms of patient safety with deficits in the quality of discharge documentation causing confusion among patients and informal caregivers [12]. In 2016, the National Audit Office reported that older people occupy 62% of hospital bed days and are the group most likely to experience delayed discharges. It also identified difficulties with hospital staff maintaining knowledge of out-of-hospital services and a lack of shared understanding of what skills are needed for good discharge planning [13]. Prolonged hospital stays can significantly impede patients’ recovery, negatively affecting anything from physical health via issues like hospital-acquired infections and increased complications due to immobility to psychological health [14]. For this reason, coordination of continued care and support at home is a growing area of focus in both research and policy making. A recent Cochrane review concluded that a structured discharge plan that is tailored to the individual patient probably brings about a small reduction in the initial hospital length of stay and readmissions to hospital for older people with a medical condition [15]. Accordingly, recent guidance on hospital discharge from the Department of Health and Social Care [16] insists that effective discharge planning can best be achieved through shared decision-making (SDM). That is, providing tailored, person-centred, strengths-based choice for individuals, who should be supported to make fully informed decisions, with input from their relatives or unpaid carers if they wish. Decisions such as care management, emergency planning, readmission risk and caregiver involvement [17].

However, despite efforts to improve the discharge planning process and subsequent outcomes, existing mechanisms fail to accurately identify older person’s needs for follow-up care [17] thus SDM remains challenging for both patients and healthcare professionals.

It is often hindered by time constraints, professionals not understanding or valuing SDM, patients lacking knowledge and confidence to participate, poor communication, and lack of trust between patients and professionals [18]. Organisational barriers include lack of formal training for healthcare professionals, busy ward environments, lack of private spaces, and absent guidance and leadership for SDM [19]. These barriers are exacerbated for frail older people facing discharge from hospital, due to patients’ cognitive or physical impairments, limited communication skills among health professionals, risk averse approaches to discharge, limited family carer engagement, and organizational barriers, such as time pressure and high patient through-put [20, 21].

Pel-Littel et al. [20] have identified that an explicit invitation to participate in SDM is important to older adults and that health professionals need a supporting organizational context and good communication skills to devise an individualized approach for patient care. A first realist synthesis of SDM literature by Waldron et al. [22] begins to address in which situations, how, why, and for whom SDM between patients and healthcare providers contributes to improved engagement in the SDM process [22]. It identifies the quality of patient and health care professional relationships, and perceptions of SDM self-efficacy for both groups, as key mechanisms promoting engagement. The difficulty of the decisions involved and organisational support were key contextual factors affecting engagement [22] therefore identifying factors influencing acceptability of SDM in populations that predominantly believe in the authority of the healthcare professional; is needed [8].

There currently remains no consensus on the best ways to achieve SDM, as the quality of evidence for interventions is minimal [11, 23, 24] with studies recommending evaluating SDM interventions and outcomes in specific care settings and with specific groups [18] using validated outcome measures that are valued by multiple stakeholders [24]. Equally, evaluating patient-centred SDM with older patients with complex needs and clearly articulating the theory underpinning the design of SDM interventions; is also needed [23]. Consequently, research is needed to develop theory-based, multi-component, tailored, acceptable, SDM interventions that will facilitate person-centred discharge planning for frail older adults (with input from their relatives or unpaid carers if they wish) and that are valuable to all stakeholders.

Waldron et al. [22] provide a welcome initial understanding into the theory behind SDM, demonstrating that SDM works in a complex manner, and for any individual patient and healthcare professional there may be an array of interconnected mechanisms at play [22]. Waldron et al. [22] developed programme theory of SDM across various healthcare contexts, decision types and patient demographics however does not specifically focus on older adults as a distinct population group. There is reference to hospital-based scenarios, particularly in oncology, however does not specifically focus on hospital discharge. Therefore, further work is needed to develop Waldron’s theory of SDM to consider what works for whom and why for frail older adults when considering discharge from hospital.

## Aims

Building on the recommendations from previous research including the work of Waldron et al., this project aims to develop a multi-component intervention to prepare health professionals, patients, and patients’ carers to engage in SDM, and to evaluate the feasibility of implementing the intervention in a health and social care trust in Northern Ireland among frail, older adults who are planning their discharge from hospital.

### Objectives

1. To conduct a realist review of the literature on interventions intended to promote SDM in relation to hospital discharge for frail older people.
2. To co-design a multi-component intervention to prepare health professionals, patients, and patients’ carers to engage in SDM.
3. To develop a programme theory to support the content and implementation of the intervention.
4. To implement and evaluate the acceptability and feasibility of the intervention in one health and social care trust in Northern Ireland.
5. To test the suitability of data collection and outcome measures.
6. To carry out a cost analysis of the intervention.

## Plan of investigation

### Study design

Our study is designed in three stages using realist methodology that aims to understand why, how, for whom, and in what context complex interventions work (or do not work) [25]. It seeks to develop theory about how interventions trigger mechanisms in specific contexts to produce their intended outcomes, often characterised as ‘context-mechanism-outcome configurations’ or ‘CMOs’ [25, 26]. The MRC guidance recognises the utility of realist evaluation to identify mechanisms of change, contextual factors, and how these combine to produce outcomes [27].

**Fig. 1:**
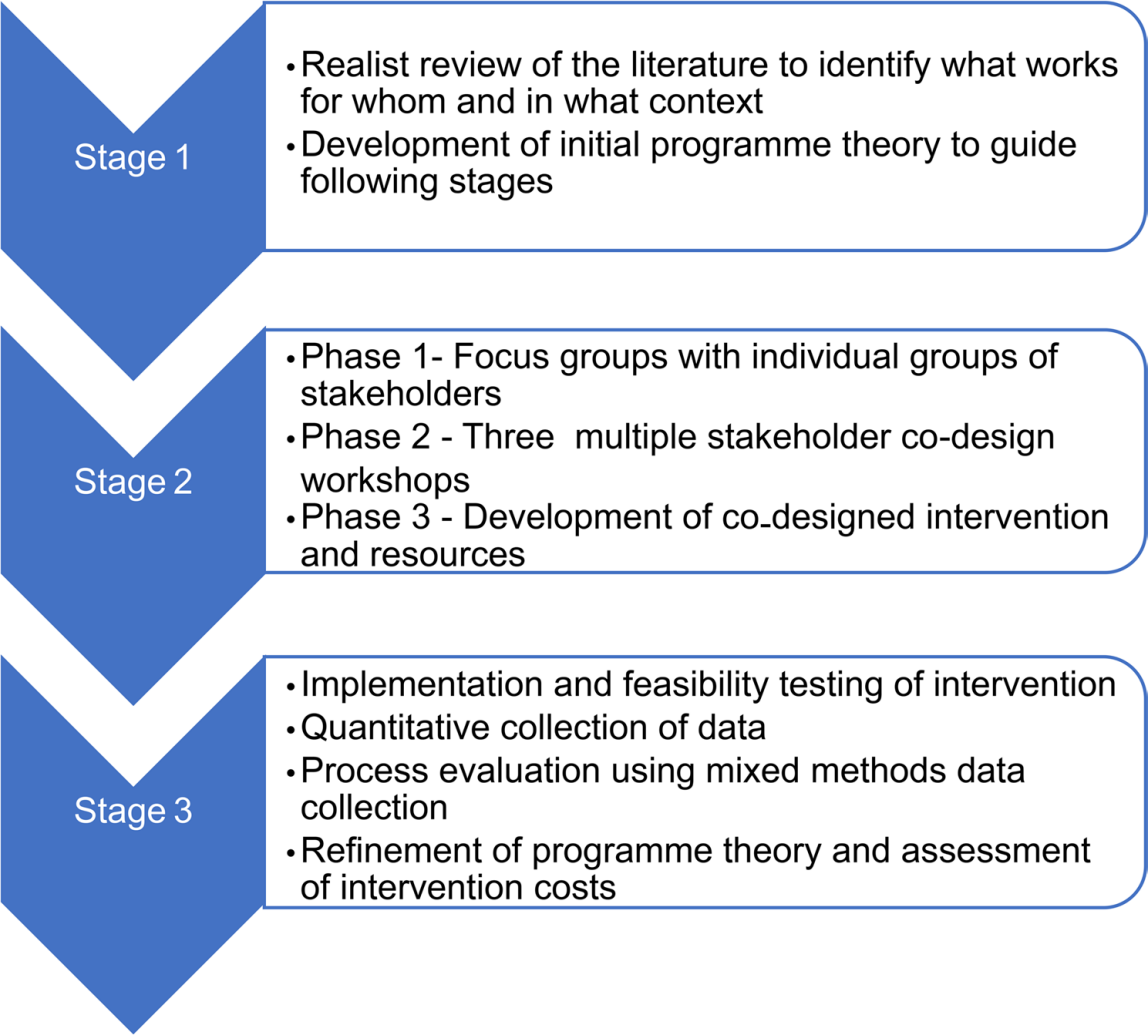
Overview of study

The three stages consist of:

1. Realist review of the literature will enable formulation of initial programme theory, including the impact of context and the identification of uncertainties.
2. Co-design of the intervention will ensure the inclusion of diverse stakeholders in the development of the intervention, with further development of the programme theory.
3. A mixed methods realist evaluation [28] of the intervention which will allow feasibility testing of implementation processes, measurement of intervention costs, testing of outcome measures, and refinement of the programme theory. The mixed methods will follow a sequential explanatory design. This consists of quantitative followed by qualitative phases. We will first collect and analyse the quantitative data to provide an overview of implementation of the intervention, then seek qualitative data to explain the quantitative findings by exploring participants’ views and perceptions of implementation in more depth [29]. (Fig. 1.)

The study design will draw on the updated Medical Research Council (MRC) guidance for developing and evaluating complex interventions [27], which considers complex intervention research in four phases: development or identification of the intervention(Stage 1 and 2), feasibility, evaluation, and implementation(Stage 3). This research will move through the first two phases: development of the intervention, and assessment of intervention feasibility (including aspects of evaluation design). At each phase, six core elements will be considered to answer the following questions:

• How does the intervention interact with its context?
• What is the underpinning programme theory?
• How can diverse stakeholder perspectives be included in the research?
• What are the key uncertainties?
• How can the intervention be refined?
• What are the comparative resource and outcome consequences of the intervention?

This study will also be guided by an Expert Advisory Group (EAG). Collaboration with Patient Public Involvement (PPI) groups has ensured that older people and carers are included as equal partners in its membership, alongside other members representing PPI, policy, education, research and practice across HSC organisations. This aligns with co-design which is a well-established methodology for service improvement in Northern Ireland. The lived experiences of older people, as expert sources of knowledge, will provide rich, in depth, qualitative understanding of the hospital discharge and shared decision-making processes. The value of careful engagement with vulnerable people to improve services has been highlighted by Mulvale and Robert [30] and has been integrated into each stage of this study. For example, the EAG will provide input into the recruitment and consent processes, development of the intervention and associated resources, engagement with analysis of data and support with dissemination. We have included PPI representation within the Expert Advisory Group and throughout the codesign through organisations such as Patient Client Council, Age Northern Ireland, Public Involvement Enhancing Research Northern Ireland.

### Methods

#### Stage 1. Realist review of the literature

A systematic realist review, following RAMESES guidelines [31], will critically examine the interaction between contexts, programme mechanisms, and outcomes in a sample of studies of SDM interventions for frail older people. This will build on and update the Waldron et al. realist review which focused on SDM more broadly [22].

The objectives of the review will be:

- 1. To identify the dominant program theories in relation to how SDM can be successfully implemented for this population.
- 2. To identify factors that may help or hinder the implementation of SDM, the Consolidated Framework For Implementation Research [32] will be used to assess potential influences on implementation thus supporting the development of the programme theory. We will focus on the following constructs:
  - The characteristics of the intervention
  - The outer and inner settings for implementation
  - The characteristics of the individuals involved
  - The implementation process
- 3. To develop a programme theory based on context-mechanism-outcome configurations to help explain how the intervention may work, so meeting the need to identify underpinning programme theory, as outlined in the MRC guidance [27].

The realist review will enable the development of initial programme theory, showing how complex SDM interventions introduce ideas and opportunities for change into existing social and organisational systems; and how people interpret and act upon these opportunities [33–35]. As part of the review, we will evaluate existing training resources for SDM and consider how or whether resource components can be adapted for our own intervention at the co-design stage. The final stage of the review process will develop a candidate programme theory.

#### Stage 2. Co-design of the intervention

Co-design is a participatory method of engaging multiple stakeholders in the development and design of services [36]. Co-design ensures all voices are heard and the end intervention meets the needs of all stakeholders [37]. Co-design has been adopted by the Department of Health NI [38] and used successfully in co- design of cancer carer services [39] and engaging service users, carers and health care practitioners in curriculum development [37]. Co-design will contribute to including diverse stakeholder perspectives, and identifying key uncertainties as recommended in the MRC guidance [27].

The co-design process will comprise three phases engaging all stakeholders: service users/carers, health professionals, and the Expert Advisory Group in each phase. We recognise that not all participants will be willing or able to attend throughout the process. Therefore, in addition to the Expert Advisory Group, we will recruit 24 participants: four nurses, four doctors, and four allied health professionals, six service users and six relatives of service users. This will ensure sufficient attendance and continuity from each group of participants across all activities, without necessitating attendance at all stages.

##### Phase 1

Focus groups with two groups of stakeholders (service users and their carers, and healthcare professionals) from Health and Social Care Trusts to ascertain their experience and perception of the shared decision-making process. The results of the realist review will act as trigger points for discussion. Each focus group will be recorded, transcribed and thematically analysed to inform Phase 2 workshops. This Phase will also identify SDM champions and service users/carers interested in the co-design process for Phase 2.

##### Phase 2

will comprise three consecutive multiple stakeholder workshops with approximately 12 service users/carers and health care professionals around the same tables. This process has worked effectively in other environments [37]. 

##### Workshop 1

Presentation of the findings from Phase 1 to act as triggers for discussion of what SDM is and what works for each group of stakeholders. For example, models of SDM from the literature which are congruent with the emerging theory may be presented and participants asked to rank these in order of preference. This may include the role of decision aids for patients and carers. The discussions will be captured via note taking and artefacts from activities within the workshop such as flip chart notes.

##### Workshop 2

Results from Workshop 1 will be presented to ensure the understanding of the researcher matches that of participants and to act as triggers discussion. Workshop 2 will focus on stakeholder perspectives of how to create enablers and overcome barriers to the SDM process to facilitate the implementation of the intervention.

##### Workshop 3

This will focus on the development of intervention resources tailored for each group of stakeholders, drawing on existing resources and the new ideas recommended in the co-design process.

##### Phase 3

Using the information from Phase 2, SDM champions and service user/carers from Phase 2 will work with the researcher to develop the intervention and associated resources/training materials building on NICE guidelines [15]. This will include working a with a communications company to develop a digital resource that reflects the perspective of all stakeholders with video and interactive elements to promote engagement with the resource. The research team have developed previous codesign resources using this approach. The Expert Advisory Group will also be invited to reflect and contribute to the resources at this stage and provide feedback.

Data will be analysed from the focus group and codesign groups against the candidate theory from the systematic realist review of the literature. Data will be used to develop existing CMO configurations or develop new configurations.

#### Stage 3. Implementation, feasibility, and costing of the intervention

### Setting

The co-designed intervention will be implemented in one inpatient medical ward in one health and social care trust in Northern Ireland.

### Intervention participants

Frail older people who are an in-patient on the intervention ward, and their carers; and nurses, doctors, allied health professionals, and social workers working with patients on the ward to plan their discharge. Frailty will be assessed using the Rockwood Clinical Frailty Scale as part of usual care [40]. Gatekeepers who know the older person will assess capacity to consent to be part of this research prior to being approached by the researcher.

### Intervention

Subject to the co-design process, it is anticipated that the SDM intervention with patients will broadly follow the “Three-talk model” recommended by NICE [8], proposes three steps: introducing choice; describing options; and helping people explore their preferences and make decisions [41]. There will be two broad elements to the training element of the intervention: SDM champions for health professionals; and an online resource to help prepare professionals, patients, and patients’ carers for engagement with SDM.

### Intervention processes

A key staff member identified as SDM champion (we anticipate one nurse)) will train as SDM Trainer using a training model developed by the research team and stakeholders as part of the co-design process. This is likely to include training in core competencies such as, eliciting values and goals of care, involving carers, and effective communication of options and risks [42]. Subject to the co-design process, it is anticipated that there will be an intentional blend of online and face to face learning modelling how to engage in SDM alongside training and coaching skills to engage others. The SDM champion will support health care professionals to gain the knowledge, skills, and confidence to facilitate shared decision making with patients. The work of SDM champion will be augmented by online resources such as video vignettes modelling helpful behaviours for health professionals, patients, and carers.

The SDM champion will introduce patients and their carers who consent to be part of the study to the online resources to help prepare them for engagement with SDM. The researcher will answer any questions the patient and their carers may have as part of the consent process. As the participants will be frail older adults, we anticipate that the carers may also support the older person in reviewing the resource as well as reviewing the resource themselves. The full details of intervention content and approach will be developed at the co-design stage. We will plan for the SDM intervention to be implemented for three months to allow it to embed and for participants to experience and understand implementation barriers and facilitators. The researcher will engage with the implementing team weekly until they are confident in the SDM process and to ensure fidelity to the intervention. A Principal Investigator (PI) in the Health and Social Care Trust will be identified and will have oversight of activity on the ward including identification and recruitment of participants.

### Baseline data

We will collect demographic data from all study participants as part of the research process. Patient data will include age, sex, ethnicity, educational level, place from which they were admitted, hospital admissions in the last six months, frailty as measured by the Rockwood Clinical Frailty Scale [40]. Deprivation will be estimated through the Northern Ireland Statistics Agency multiple deprivation area statistics website, using a patient’s postcode [43].

## Outcomes

Overall outcome evaluation will be guided by the ‘Quintuple Aim’ framework [44]. This is a recent development of the Quadruple Framework [45], adding ‘advancing health equity’ to the four aims of seeking to enhance patient experience, improve health, reduce costs, and improve the work life of healthcare professionals. Nundy et al. [44] emphasize the lack of health equity around the world for socially marginalized populations, including older adults, highlighting that the challenge since COVID-19 is translating this heightened social consciousness into action, particularly in communities, clinics, and health systems [44]. In line with recommendations from the MRC guidance [27], we will also seek insights to help refine the SDM intervention.

We will record recruitment, retention, and participation rates for all participants; together with the time needed to train staff, collect and analyse data. We will assess the suitability and timing of the implementation period, outcome measures, and survey instruments.

The evaluation of the SDM intervention will follow a model proposed by Muller et al. [24], based on Kirkpatrick’s four-level training evaluation model [46]. This recommends assessing the reactions of participants (in this case patients, their carers, and healthcare professionals) to the intervention; the learning they report; their behaviour in relation to SDM; and the outcomes experienced by patients, their carers, and the healthcare professionals.

Participants’ reactions to the SDM model will be captured by assessing the acceptability, appropriateness, and feasibility of the intervention components, using the Acceptability of Intervention Measure (AIM), Intervention Appropriateness Measure (IAM), and Feasibility of Intervention Measure (FIM). These are each short, four-item measures with scale values ranging from 1 (completely disagree) to 5 (completely agree) [47]. Learning will be assessed by measuring knowledge gain, attitudes, and confidence to engage in SDM, using multiple choice and open questions based on the contents covered in training.

SDM behaviour will be assessed using standardised reports from healthcare professionals, patients and their carers [24]. Lack of evidence limits consensus on the best ways to measure SDM [48, 49]. However, two widely used and partially validated self-report measures that are recommended for the National Health Service [50], are the SDM-Q-9 for patients (which will be adapted for their carers) [51], and the SDM-Q-DOC for healthcare professionals [52]. These are nine-item measures, with scale values ranging from 1 (completely disagree) to 6 (completely agree). Observation of SDM conversations will form part of the intervention for patients will be analysed by the researcher, both as part of the process evaluation (see below) and more formally as a measure of SDM using the OPTION5 observer tool [53]. 

Investigation of outcomes experienced by patients, their carers, and the healthcare professionals will be informed by the realist review and co-design stages. The realist review will identify key outcomes from the literature for frail older adults on discharge and be presented as the first phase in the co-design process. This will enable the researcher and team to identify relevant outcomes for each stakeholder group as part of the development of the intervention. Subject to the co-design process, it is anticipated that relevant measures may include the following:

For patients and carers:

- a) Patient and carer reported involvement in SDM.
- b) The SURE 4-item screening test for decisional conflict [54].
- c) 5 item Decision Regret Scale [55].
- d) Length of hospital stay.
- e) Discharge destination.

For healthcare professionals:

- a) Willingness to incorporate shared decision making into practice will be measured using the incorpoRATE tool [56, 57].
- b) We will also explore the impact of the intervention on work life in interviews as part of our process evaluation.

On completion of data collection, each measure will be assessed for suitability.

### Process evaluation

The process evaluation will be informed by the implementation theory developed through the realist review and refined at the co-design stage for the intervention. It will also enable us to address how the intervention interacts with its context, as recommended by MRC guidance [27]. This will involve:

• Observation of the SDM training for SDM Champions.
• Towards the end of the three-month intervention period, developing a process map [58] of the personnel and systems involved in managing the SDM intervention with the help of key healthcare professionals, SDM Champions, administrators and managers.
• Two focus groups with healthcare professionals, SDM Champions, administrators, and managers using the intervention, plus interviews with six patients and six carers who have used the SDM intervention. These will use a semi-structured guide designed to elicit their experience of using the intervention (what they hoped to achieve, their concerns and feelings) and their views on the barriers and facilitators of implementation. In line with the Quintuple Aim framework, we will also seek insight on whether the intervention has enhanced patient experience and improved the work life of the healthcare professionals. Participating patients and carers will be asked before discharge if they are willing to engage in an interview.
- Using the CMO configurations and developing programme theory, data will be analysed to add to/refine the CMO configurations until a final programme theory has been developed.

### Realist synthesis

At each stage of data collection, through a process of discussion and consensus building we will seek to further develop our programme theory in relation to the implementation of the intervention. Drawing on the insights provided by different stakeholders in the three phases of data collection, we will consider each piece of data, assessing the degree to which it supports, refutes, or modifies developing theory, thus building our understanding of how the intervention may work.

### Costing the intervention

Costing the intervention will begin the process of comparing resource and outcome consequences, as recommended in MRC guidance [27]. We will record training time per participant and ask healthcare professionals to record the time spent on SDM, including time spent introducing SDM, and all conversations and interactions with colleagues and carers that focus on reaching a joint decision about care. From this data we will estimate costs per hour for healthcare professionals. This will also allow us to estimate the costs in time to patients and time and travel for carers. We will seek to distinguish SDM from activities related to implementing the decisions arising from SDM in relation to patient discharge in itself.

## Discussion

This research will produce a theory-based, multi-component, tailored, acceptable, SDM intervention intended to facilitate person-centred discharge planning for frail older adults, with input from their relatives if they wish. It will also test the suitability of outcome measures, estimate the costs of the intervention, and prepare the way for formal piloting and evaluation of the intervention, as recommended by the Medical Research Council guidance. Such interventions have the potential to positively impact frail older people, relatives/carers and health professionals whilst providing savings in resources for the health and social care services.

The aforementioned RAMESES guidelines [31] will enable consistency and rigor of reporting to ensure that findings are clearly understood through explicit articulation of iterative programme theory development.

### Strengths and limitations

Co-design of the intervention will ensure the inclusion of diverse stakeholders in the development of the intervention, with further development of the programme theory. The co-designed intervention will be implemented in one inpatient medical ward. A realist approach explores not only if an intervention is successful or unsuccessful but why this is the case. Therefore, this provides the authors with a unique opportunity to enhance practice through their research findings.

### Final dissemination event

As service user and their relatives have been engaged at every stage of this project, we will host a half- day PPI conference in partnership with the Expert Advisory Group and our advocacy groups. The SDM model, training and results of the evaluation will be shared at a regional event. We will invite our PPI representatives to speak about their role in the project and to outline the impact they believe the project will have on patient care. Attendees will be asked for their feedback on the SDM intervention to contribute to future work.

## Data Availability

Not applicable.

## Abbreviations

CMO: Context-mechanism-outcome
NICE: National Institute for Health and Care Excellence
PPI: Patient and Public Involvement
PROSPERO: International Prospective Register of Systematic Reviews
RAMESES: Realist and Meta-narrative Evidence Syntheses Evolving Standards
SDM: Shared Decision-Making
HSC PHA R&D Division: Health and Social Care, Public Health Agency, Research and Development Division
